# Supplementary material for: Dual Role of ACBD6 in the Acylation Remodeling of Lipids and Proteins
Source: Biomolecules. 2022 Nov 22;12(12):1726. doi: 10.3390/biom12121726 (PMC9775454; doi:10.3390/biom12121726)
Supplement: Supplementary file 1 [file biomolecules-12-01726-s001.zip › biomolecules-1984764-supplementary.pdf]

Validation and Invalidation of Chemical Probes for the Human *N*-myristoyltransferases

Wouter W. Kallemeijn,[1,4] Gregor A. Lueg,[1,2,4] Monica Faronato,[1,2] Kate Hadavizadeh,[1] Andrea Goya Grocin,[1] Ok-Ryul Song,[2] Michael Howell,[2] Dinis P. Calado,[2,3] and Edward W. Tate[1,2,5,*]

[1]Department of Chemistry, Imperial College London, Molecular Research Science Hub, 80 Wood Lane, London W12 0BZ, UK

[2]The Francis Crick Institute, 1 Midland Road, London NW1 1AT, UK

[3]Peter Gorer Department of Immunobiology, School of Immunology & Microbial Sciences, King's College London, London SE1 9RT, UK

[4]These authors contributed equally

[5]Lead Contact

*Correspondence: e.tate@imperial.ac.uk

https://doi.org/10.1016/j.chembiol.2019.03.006

Snapshot of Figure 2, panel B and D. Effects on Cellular N-myristoylation, N-myristoyltransferases, and Substrate ARL1 in Living Cells

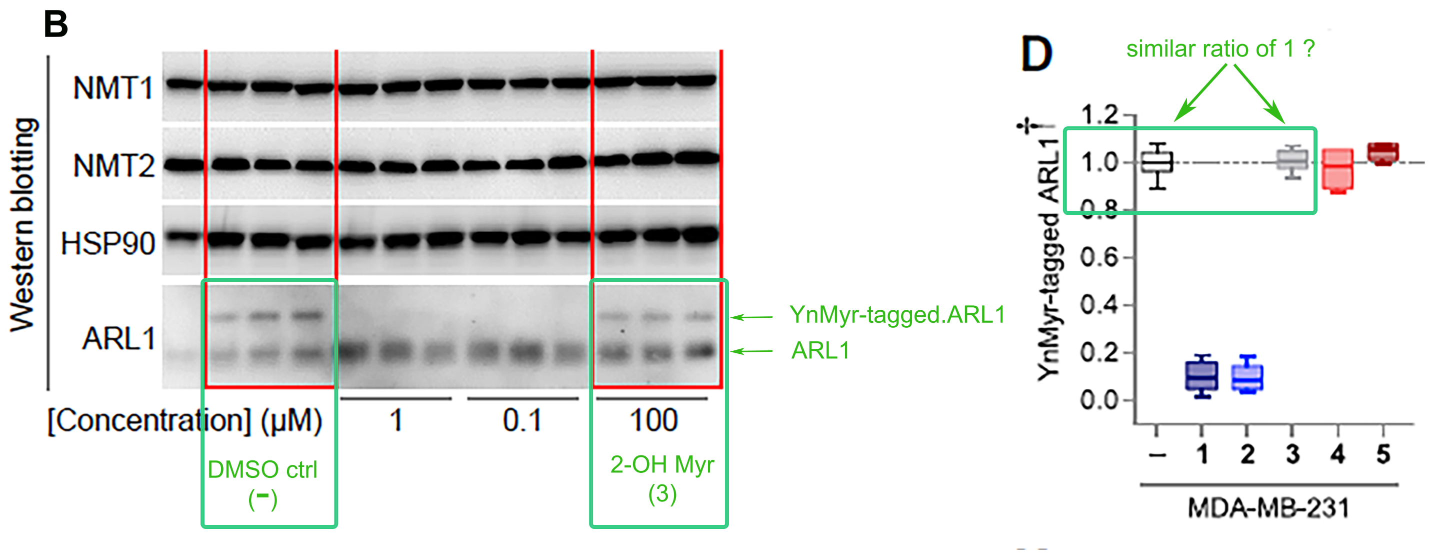

(Additions by Soupene et al are indicated in green colored text)
